# RETRACTION: Innate Immune Molecule Surfactant Protein D Attenuates Sepsis‐Induced Acute Kidney Injury Through Modulating Apoptosis and Nfκb‐Mediated Inflammation

**DOI:** 10.1111/iwj.70636

**Published:** 2025-04-23

## Abstract

**RETRACTION:** LuS.‐J. , XuJ.‐H. , HeZ.‐F. , WuP. , NingC. , and LiH.‐Y. , “Innate Immune Molecule Surfactant Protein D Attenuates Sepsis‐Induced Acute Kidney Injury Through Modulating Apoptosis and Nfκb‐Mediated Inflammation,” International Wound Journal 17, no. 1 (2020): 100–106, 10.1111/iwj.13237.31701658 PMC7949018

The above article, published online on 08 November 2019, in Wiley Online Library (http://onlinelibrary.wiley.com/), has been retracted by agreement between the journal Editor in Chief, Professor Keith Harding; and John Wiley & Sons Ltd. Following an investigation by the publisher, all parties have concluded that this article was accepted solely on the basis of a compromised peer review process. In addition, further investigation by the publisher found that the authors provided incomplete ethical approval information for animal experiments performed in the study. The editors have therefore decided to retract the article. The authors did not respond to our notice regarding the retraction.

**RETRACTION:** S.‐J. Lu , J.‐H. Xu , Z.‐F. He , P. Wu , C. Ning , and H.‐Y. Li , “Innate Immune Molecule Surfactant Protein D Attenuates Sepsis‐Induced Acute Kidney Injury Through Modulating Apoptosis and Nfκb‐Mediated Inflammation,” International Wound Journal 17, no. 1 (2020): 100–106, 10.1111/iwj.13237.31701658 PMC7949018

The above article, published online on 08 November 2019, in Wiley Online Library (http://onlinelibrary.wiley.com/), has been retracted by agreement between the journal Editor in Chief, Professor Keith Harding; and John Wiley & Sons Ltd. Following an investigation by the publisher, all parties have concluded that this article was accepted solely on the basis of a compromised peer review process. In addition, further investigation by the publisher found that the authors provided incomplete ethical approval information for animal experiments performed in the study. The editors have therefore decided to retract the article. The authors did not respond to our notice regarding the retraction.

